# Survey of Hypoglycemia in Elderly People With Type 2 Diabetes Mellitus in Japan

**DOI:** 10.14740/jocmr2340w

**Published:** 2015-10-23

**Authors:** Masahiro Fukuda, Kunihiro Doi, Masahiro Sugawara, Yoshikazu Naka, Kouichi Mochizuki

**Affiliations:** a Fukuda Clinic, 2F Shin Osaka Brick Building, 1-6-1 Miyahara, Yodogawa-ku, Osaka-shi, Osaka 532-0003, Japan; b Doi Clinic, 1-54 Todou Aramaki, Uji-shi, Kyoto 611-0013, Japan; c Sugawara Clinic, 3-9-16 Shakujiimachi, Nerima-ku, Tokyo 177-0041, Japan; d Tomei-Atsugi Hospital, 232 Funako, Atsugi-shi, Kanagawa 243-8571, Japan; e Mochizuki Naika Clinic, 1F Katsura Heights, 4-5 Aioicho, Itabashi-ku, Tokyo 174-0044, Japan; f Member of the Japan Physicians Association.

**Keywords:** Hypoglycemia, Hypoglycemic symptom, Hidden hypoglycemia, Knowledge of hypoglycemia, T2DM, Elderly, Medication, Countermeasure

## Abstract

**Background:**

The number of elderly type 2 diabetes mellitus (T2DM) patients in Japan is increasing continuously. Hypoglycemia is a significant issue in their treatment. However, the actual situation and related details of their hypoglycemia remain unclear. In order to elucidate them, the Japan Physicians Association conducted a large-scale questionnaire survey for physicians and their outpatients all over Japan.

**Methods:**

Targeted elderly T2DM outpatients were 65 years old or older in 2011. Specialized questionnaire survey forms were distributed to both of physicians and patients. The forms for physicians included questions whether patient had hypoglycemia in the last 1 month or 1 year; those for patients included whether they experienced it in the same durations and any of the 28 symptoms that are suggestive of hypoglycemia or pertaining to geriatric syndrome in the last 1 month, as well as questions about knowledge regarding hypoglycemia. We analyzed associations between hypoglycemia and the symptoms, and between hypoglycemia and medications.

**Results:**

Of 15,892 T2DM patients (age, 74.2 ± 6.3 years; diabetes duration, 12.8 ± 8.9 years; HbA1c, 7.0±1.0%), dipeptidyl peptidase-4 inhibitor (DPP-4i) was the most prescribed medication among all oral hypoglycemic agents (OHAs). The frequencies of hypoglycemia in the last 1 month recognized by physicians and experienced by patients were 7.8% and 10.4% (P < 0.0001), and in the last 1 year were 15.5% and 21.1% respectively (P < 0.0001). The most common symptom was “weakness, fatigue/feeling languid” and the majority of all patients reported neuroglycopenic or autonomic symptoms. Regarding monotherapy, hypoglycemia was observed in 32.7% of the patients with insulin, 4% in sulfonylurea (SU), 3.8% in glinide, and 3.5% in pioglitazone. The questions asking knowledge about hypoglycemia revealed that SU or insulin users had significantly more knowledge of hypoglycemia than others (P < 0.001); however, 63% of patients using insulin, and 31% of patients using SU always carried glucose or a similar medication with them.

**Conclusions:**

The present study suggested two types of “hidden hypoglycemia”, one is that physicians did not detect and the other one is that patients were not aware. It is vital that physicians strive to prevent hypoglycemia by paying closer attention to symptoms of “hidden hypoglycemia” in their elderly patients.

## Introduction

The number of patients with type 2 diabetes mellitus (T2DM) in Japan has been increasing. According to the International Diabetes Federation (IDF) Diabetes Atlas [1], the number of patients with diabetes aging 20 - 79 years in 2014 was 7.21 million in Japan. Furthermore, it is estimated that additional 3.89 million undiagnosed patients with suspected diabetes exist. Hence, Japan is ranked 10th worldwide in terms of its diabetic population. In particular, the population is progressively aging in Japan [2]; thus, the proportion of elderly individuals in patients with diabetes is higher compared with other countries. Moreover, according to the 2014 National Health and Nutrition Survey, increasing patients in those with diabetes were the elderly patients in Japan, and it will continue to increase.

Hypoglycemia is a significant issue in treatment of diabetes for the elderly patients. Hypoglycemia worsens quality of life (QOL) and reduces quality-adjusted life years (QALY) [3]. In particular, elderly individuals also experience symptoms caused by aging as follows: decreased activities of daily living, lowered physiological function of the liver and kidneys, and decreased cognitive function. Thus, elderly patients are more likely to develop iatrogenic hypoglycemia than non-elderly patients. In addition, hypoglycemic symptoms change with age and those symptoms are often atypical expression in elderly individuals [4]; therefore, treatment for their hypoglycemia tends to be delayed and increasing disease severity. According to the report regarding severe hypoglycemia such as hypoglycemic coma in emergency visit [5], three major factors are advanced age, renal function degeneracy, and sulfonylurea (SU) agent or insulin use [6]. Furthermore, it was reported that hypoglycemia decreases QOL of elderly individuals [7], due to decreased cognitive function [8, 9], exacerbation of depression symptoms [10], and increased risk of bone fracture accompanying falls [11]. Moreover, decreased QOL in elderly individuals increases the risk for cerebral stroke [12].

Thus, hypoglycemia is a significant problem in the treatment of elderly patients with diabetes; however, related details remain unclear. Therefore, to better elucidate the state of elderly T2DM patients, patient’s pathophysiology, treatment, and hypoglycemia status in Japan, the Japan Physicians Association conducted the “Fact-finding Survey on Elderly Type 2 Diabetes Patients Undergoing Outpatient Treatment,” a large-scale questionnaire survey of physicians and their outpatients at age of 65 years or older with T2DM receiving treatment at medical institutions all over Japan, and included members of the Japan Physicians Association. Based on the results of the study, the present report focused on the feature of hypoglycemia in elderly patients with T2DM in Japan.

## Methods

**Figure 1:**
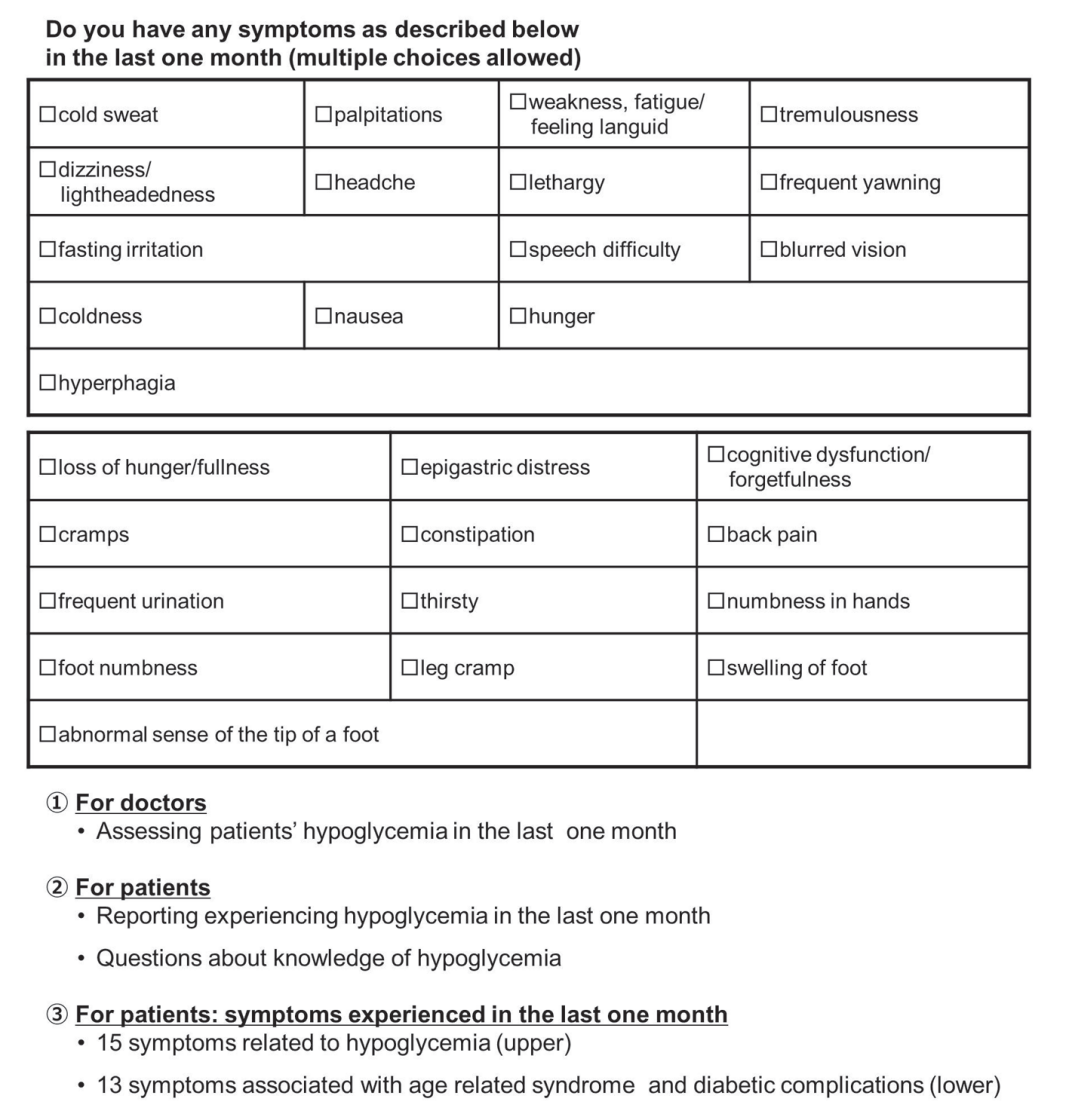
Questionnaire of hypoglycemia. The items were made in reference to a chapter of hypoglycemia in MEDSi Joslin Diabetes.

The “Fact-finding Survey on Elderly Type 2 Diabetes Patients Undergoing Outpatient Treatment” targeted T2DM patients aging ≥ 65 years and receiving treatment as an outpatient in 2011. In this study, to prevent bias related to regional localities and treatment policies of particular physicians, the target number of enrolled patients was set as 10,000. Then data were collected through the cooperation of nationwide medical institutions (mainly clinics) where the internal review board was carried out at each institution, and written informed consent was obtained from each participant. All data identifiable to each participant are not disclosed. Specialized survey form was distributed to physicians, who filled out items regarding the targeted patients. Survey items included patient age, disease duration, HbA1c, blood glucose levels, presence of diabetes complications, type of medication currently prescribed for diabetes, and whether the targeted patient had hypoglycemia in the last 1 month or 1 year. Hypoglycemia did not necessarily require measurement of blood glucose levels, but rather was based on the physician’s judgment while considering the medical interview results. A questionnaire was simultaneously administered to all patients with the physicians’ survey. The questionnaires for patients were as follows: 1) the presence or absence of symptoms in the previous month (15 items focusing on symptoms suggestive of hypoglycemia and 13 items pertaining to geriatric syndrome). These survey items were chosen in reference to Joslin’s Diabetes Mellitus, 14th edition [13]; 2) knowledge regarding hypoglycemia (Do you know what hypoglycemia is?); 3) knowledge regarding hypoglycemia, such as countermeasures for hypoglycemia, symptoms, causes, and susceptible periods in a day; 4) whether the patient had experienced symptoms suspected as hypoglycemia in the last 1 month; 5) whether the patient had experienced symptoms suspected as hypoglycemia in the last 1 year; and 6) whether the patient carried glucose or an equivalent item as a countermeasure for hypoglycemia. In statistical analyses, the Chi-square test was used to compare frequencies (e.g., yes or no) between two groups, whereas the Cochran-Armitage trend test was performed for three groups or more, and logistic regression analysis was applied to identify the association between hypoglycemia and symptoms experienced by patients, and between hypoglycemia and medications (Fig. 1).

## Results

Responses were received from 15,892 T2DM patients (male 52.5%, female 47.5%) aging 74.2 ± 6.3 years (range, 65 - 100 years), with duration of diabetes of 12.8 ± 8.9 years, HbA1c of 7.0±1.0%, and body mass index (BMI) of 24.1 ± 3.7 kg/m^2^. Diabetes-related complications in 16.0% patients were retinopathy, in 26.3% were nephropathy (stage 2 or higher), and in 20.5% were neuropathy. In addition, 15.9% and 10.5% patients had a history of cardiovascular disturbance and cerebrovascular disease, respectively (Table 1).

**Table 1:** Clinical Characteristics of Elderly T2DM Patients

	Mean ± SD	N
Registration patients, n		15,892
Mean age, years	74.2 ± 6.3	15,892
Gender, females (%)	7,516 (47.5)	15,835
Mean height, cm	157.2 ± 9.2	14,886
Mean weight, kg	59.8 ± 11.1	15,057
Mean BMI	24.1 ± 3.7	14,681
Diabetes		
Mean disease duration, years	12.8 ± 8.9	
Mean onset age, years	61.4 ± 10.0	
Mean fasting blood sugar level, mg/dL	128.7 ± 34.1	3,562
Mean HbA1c, % (NGSP)	7.0 ± 1.0	15,426
Diabetic retinopathy, n (%)	2,424 (16.0)	15,114
Diabetic nephropathy, n (%)	3,995 (26.3)	15,206
Diabetic neuropathy, n (%)	3,091 (20.5)	15,064
Medical history		
Cardiovascular disease, n (%)	2,278 (15.9)	
Cerebrovascular disease, n (%)	1,501 (10.5)	

### Prescriptions of oral hypoglycemic agents (OHAs) for elderly diabetes patients

**Figure 2:**
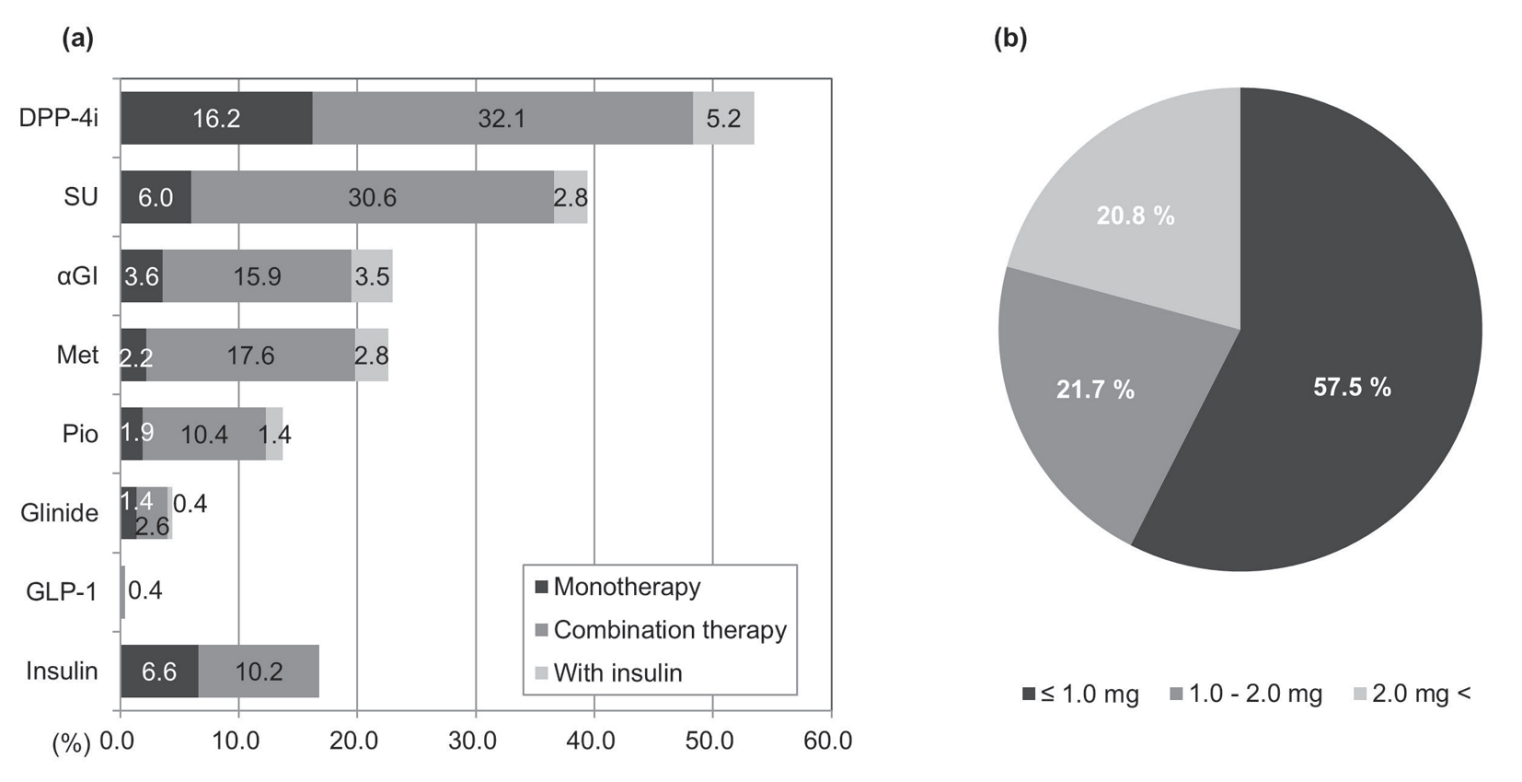
Trends in antidiabetic drug prescriptions. (a) Proportions of patients who were taking any one of eight drugs (n = 15,855). Met: metformin; Pio: pioglitazone. (b) Patient SU dosage/day (n = 5,825). Conversion rate: glimepiride 1 mg = gliclazide 40 mg = glibenclamide 0.625 mg.

Figure 2a shows the frequency of prescriptions for medications. The highest prescription rate for OHAs was for dipeptidyl peptidase-4 (DPP-4) inhibitors, which were prescribed to over 50% of patients (monotherapy, 16.2%; combined therapy, 37.3%). The second highest prescription rate was for SU agents (monotherapy, 6.0%; combined therapy, 33.4%), followed by alpha-glucosidase inhibitors (α-GIs; monotherapy, 3.6%; combined therapy, 19.4%), metformin (monotherapy, 2.2%; combined therapy, 20.4%), pioglitazone (monotherapy, 1.9%; combined therapy, 11.8%), and glinide agents which were less commonly prescribed (monotherapy, 1.4%; combined therapy, 3%). Regarding the type of SU agents used, 80.7% of prescriptions were for glimepiride, 8.8% for gliclazide, and 10.5% for glibenclamide (not shown in the figure). A 1-day dosage was converted as follows: 1 mg of glimepiride = 40 mg of gliclazide = 0.625 mg of glibenclamide.

Using this formula to convert results for glimepiride, the most commonly administered SU agent, we observed that 1.0 mg/day or less was administered to 57.5% patients, 1.0 - 2.0 mg/day was administered to 21.7%, and > 2.0 mg/day was administered to 20.8%. With the mean dosage of 1.94 mg/day, the amounts of SU prescription were relatively low (Fig. 2b).

Regarding injection therapy, GLP-1 agonists were administered to 0.4% patients, whereas insulin therapy was administered to 16.8% of patients (Fig. 2a).

### State of hypoglycemia

**Figure 3:**
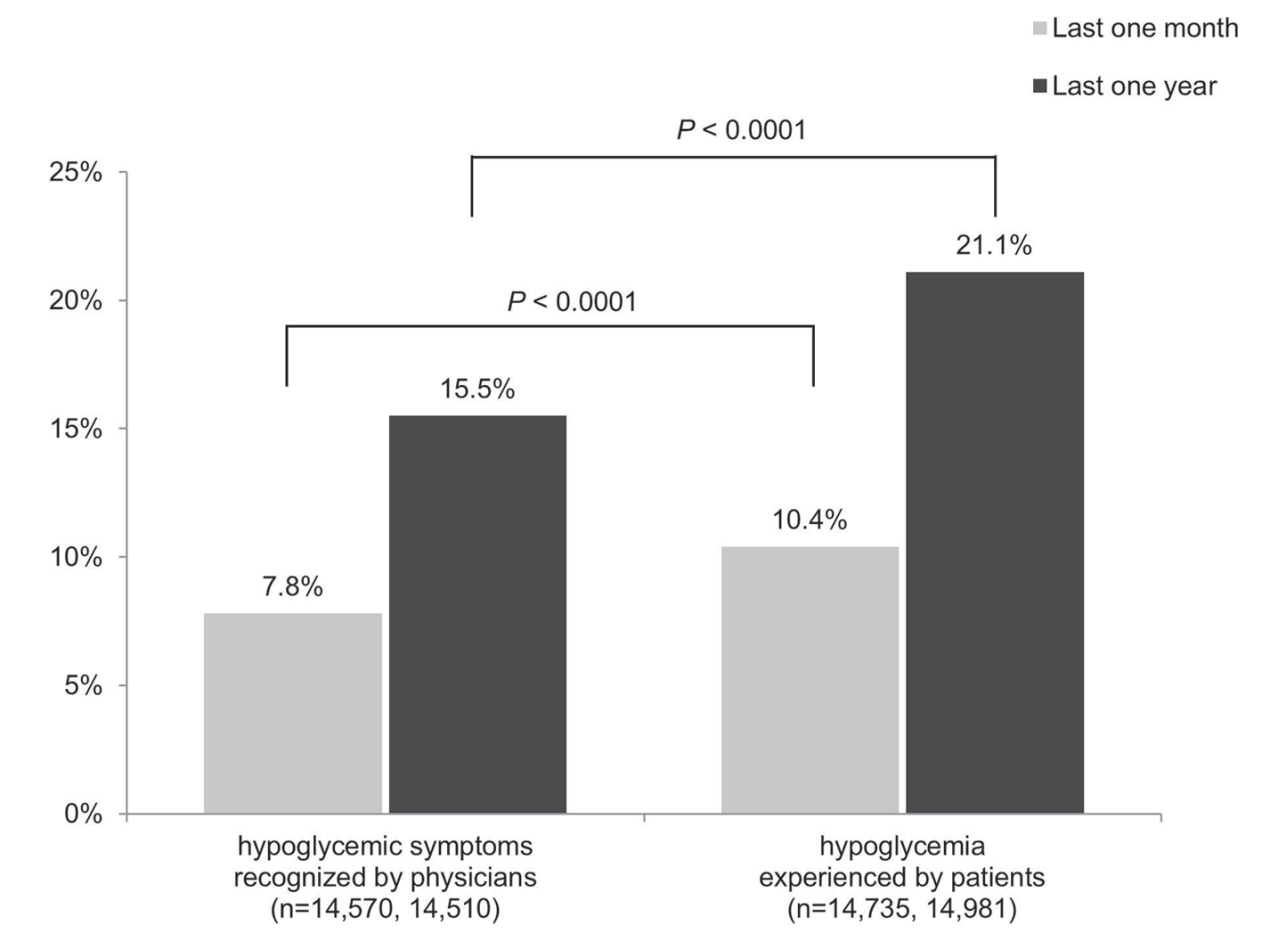
State of hypoglycemia.

As shown in Figure 3, the overall frequency of patients’ hypoglycemia that physicians recognized in the last 1 month was 7.8%. This significantly differed from patient questionnaire results, in which 10.4% of patients had experienced hypoglycemia in the last 1 month (P < 0.0001). The rates of recognition for hypoglycemia over the last 1 year also significantly differed: 15.5% and 21.1% for physicians and patients, respectively (P < 0.0001). When insulin treatment was excluded, which is not shown in the figure, the frequencies of hypoglycemia for the last 1 month and the last 1 year were 3.8% and 8.8%, respectively, according to the physicians, and 6.1% and 13.8%, respectively, according to the patients.

**Figure 4:**
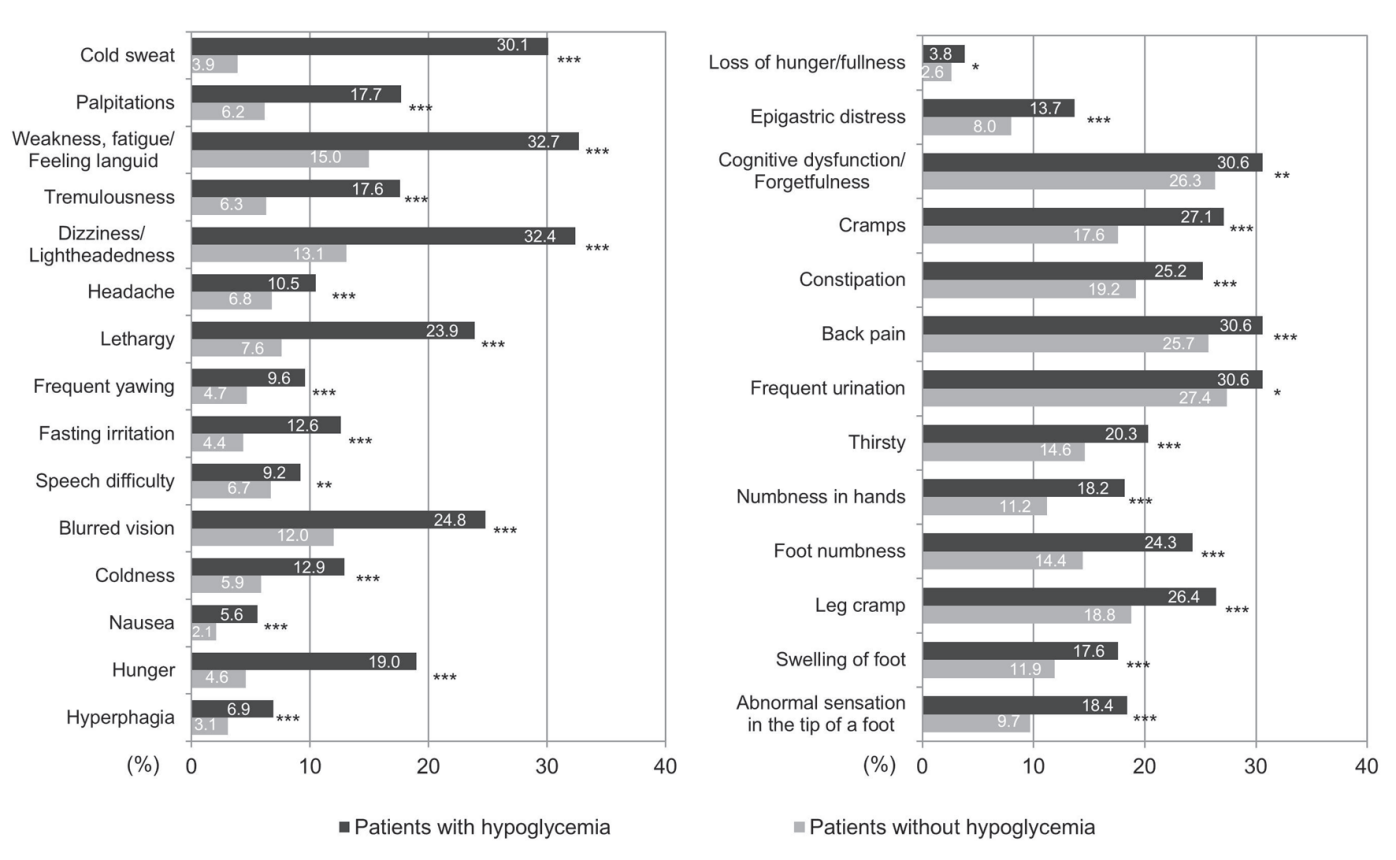
Hypoglycemia in patients with the 28 symptoms. Patients with hypoglycemia commonly presented with weakness, fatigue/feeling languid, dizziness/lightheadedness, cognitive dysfunction/forgetfulness, back pain, and frequent urination, and cold sweats. *P < 0.05, **P < 0.01, ***P < 0.001.

Regarding symptoms experienced subjectively by patients in the last 1 month, Figure 4 shows results with patients divided into groups according to whether they answered that they did or did not experience hypoglycemia. The most common symptom, “weakness, fatigue/feeling languid,” was noted in 32.7% patients in the hypoglycemia group, whereas it was noted only in 15.0% patients in the non-hypoglycemia group. Furthermore, in the hypoglycemia and non-hypoglycemia groups, “dizziness/lightheadedness” was experienced by 32.4% vs. 13.1%, respectively, followed by “cognitive dysfunction/forgetfulness” by 30.6% vs. 26.3%, “back pain” by 30.6% vs. 25.7%, “frequent urination” by 30.6% vs. 27.4%, and “cold sweat” by 30.1% vs. 3.9%, respectively. Thus, neuroglycopenic symptoms such as “weakness, fatigue/feeling languid,” “dizziness/lightheadedness,” and “cognitive dysfunction/forgetfulness” were most common, whereas among the six most experienced symptoms, “cold sweat” was the only autonomic symptom noted.

**Figure 5:**
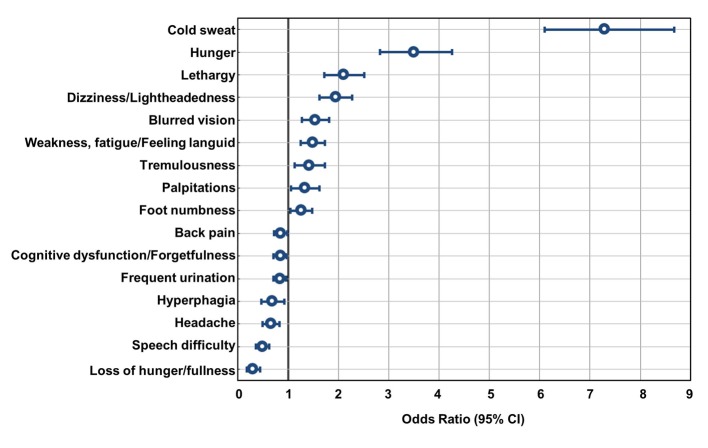
Associations between hypoglycemia and the symptoms. Multiple logistic regression analysis of the incidence of hypoglycemia by doctors' records in the last 1 month and the 28 self-reported symptoms by patients is shown above. “Cold sweat” and “hunger” indicated a strong positive relationship with the incidence of hypoglycemia. On the other hand, we found some symptoms like “loss of hunger/fullness” showed negative association with the incidence of hypoglycemia.

Many of the above symptoms were also noted by patients without hypoglycemia. We also performed multivariate analysis to assess the relationship between the absence or presence of hypoglycemia and symptoms of hypoglycemia (Fig. 5). Results indicated that “cold sweat” had the highest odds ratio (OR) of 7.4, followed by “hunger,” “lethargy,” “dizziness/lightheadedness,” “blurred vision,” “weakness, fatigue/feeling languid,” “tremulousness,” “palpitations,” and “foot numbness.” These symptoms appear to have some correlation with the presence or absence of hypoglycemia (Fig. 5). For “cold sweat” and “hunger,” both parasympathetic nervous system symptoms, the strongest and second strongest correlations were observed, respectively. These were followed by symptoms originated from glucose deficiency in the central nervous system, and unidentified complaints. Then, “palpitations” and “tremulousness,” which are symptoms originating in the sympathetic nervous system, had the lowest incidences. The ORs were low for “cognitive dysfunction/forgetfulness” and “headache,” which are categorized as central nervous system hypoglycemic symptoms. In our examination whether there were any characteristics of the symptoms observed when groups were stratified by age, such as the oldest old, symptoms such as “dizziness/lightheadedness” and “weakness, fatigue/feeling languid” was significantly more common as the elderly patients aged. However, “cold sweating” tended to decrease in those aging ≥ 80 years although it was not significant (Table 2).

**Table 2:** Hypoglycemia Symptoms and Age (Total N = 1,142)

	65 - 70 (n = 312)	70 - 75 (n = 327)	75 - 80 (n = 298)	≥ 80 (n = 205)	P value*
Cold sweat	32.1	31.2	31.2	24.1	0.09
Dizziness/lightheadedness	27.6	31.5	34.6	41.4	0.01
Weakness, fatigue/feeling languid	29.5	29.1	37.2	33.8	0.02
Blurred vision	22.4	25.4	26.8	26.2	0.46
Lethargy	24.7	20.2	21.8	31.7	0.13

*Cochran-Armitage trend test.

### Factors associated with the incidence of hypoglycemia

We investigated the relationship between the use of OHAs and incidence of hypoglycemia. In the 1,140 patients who were recognized by a physician to have experienced hypoglycemia in the last 1 month and were using diabetes medication as a monotherapy, the highest incidence of hypoglycemia was observed in the patients undergoing insulin monotherapy (32.7%). With regard to monotherapy with OHAs, incidence of hypoglycemia was high in the patients using drugs to promote insulin secretion as 4% for SU agents and 3.8% for glinide agents, followed by 3.5% for pioglitazone, whereas ≤ 2% in patients using metformin, DPP-4 inhibitors, or α-GIs. No significant differences were detected in the incidence of hypoglycemia between the patients with glinide agents and with other OHAs; however, the incidence of hypoglycemia was significantly higher in patients using SU agents than those using metformin, DPP-4 inhibitors, or α-GIs (P = 0.0257, P = 0.0001, and P = 0.0301, respectively).

**Figure 6:**
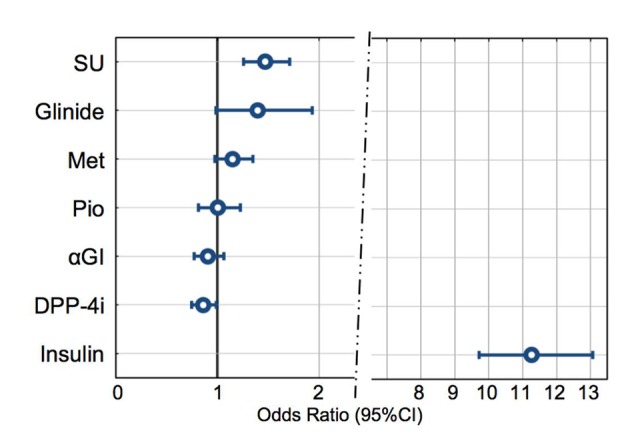
Associations between hypoglycemia and drugs. A multiple logistic regression analysis was performed between the incidence of hypoglycemia in the last 1 month, as assessed by doctors, as the dependent variable to analyze the associations with drug use. SU: significantly high OR (odds ratio). Glinide, Met, Pio, αGI: not significant. DPP-4i: the lowest OR (< 1). Insulin: prominently high OR. GLP-1 and other drugs were excluded from this analysis due to the lower number of patients using the drugs.

We also performed multivariate analysis to assess the relationship between the use of antidiabetic agents, including combined therapy, and incidence of hypoglycemia. As shown in Figure 6, the incidence of hypoglycemia was highest in patients receiving insulin therapy (OR 11.4). In the OHAs, SU agents exhibited a significantly higher incidence of hypoglycemia (OR 1.47), demonstrating that hypoglycemia was high in patients using SU agents. The OR for glinide agents was relatively high as 1.29 but did not show statistical significance. No statistically significant differences were noted for metformin, pioglitazone, or α-GIs. DPP-4 inhibitors showed OR less than 1 as 0.86, which was statistically significant; this suggested that incidence of hypoglycemia was low in patients using DPP-4 inhibitors.

**Figure 7:**
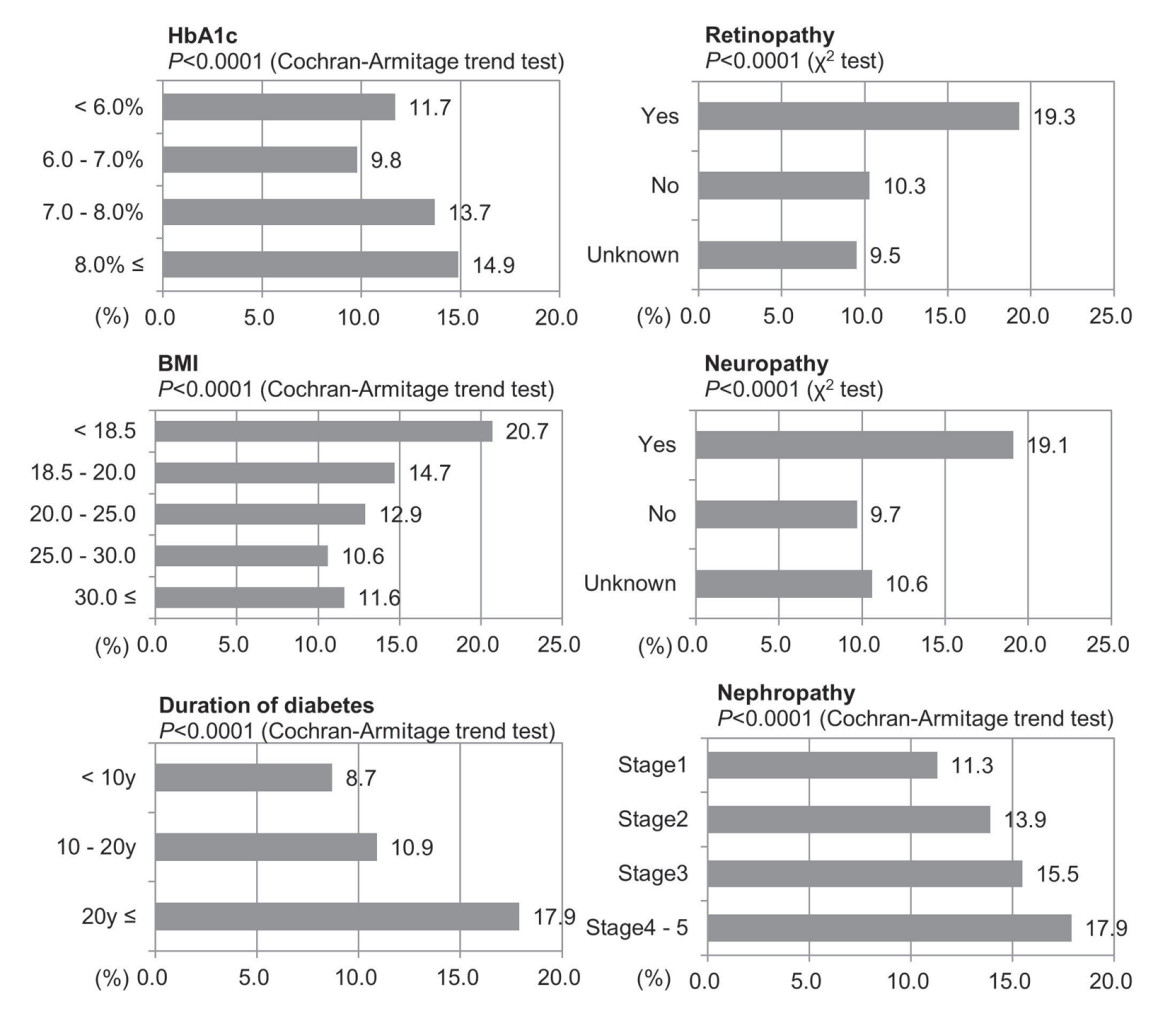
Hypoglycemia in the patients using SU or insulin. Percent of incidence of hypoglycemia based on doctors’ records.

The relationship between the number of patients who had experienced hypoglycemic symptoms recognized by a physician and HbA1c, BMI, duration of diabetes, and three major complications of diabetes were investigated (Fig. 7). Results for HbA1c indicated that patients with poorer blood glucose control, lower BMI and longer disease duration tended to be more likely to have experienced hypoglycemia (P < 0.0001).

Patients with complications of retinopathy or neuropathy had a significantly higher incidence of hypoglycemia than those without such complications (both, P < 0.0001). Similarly, patients with nephropathy were significantly more likely to have experienced hypoglycemia than those without nephropathy (stage 1 as no nephropathy). As the degree of nephropathy worsened, this trend intensified (P < 0.0001).

### Patients’ knowledge regarding hypoglycemia

**Figure 8:**
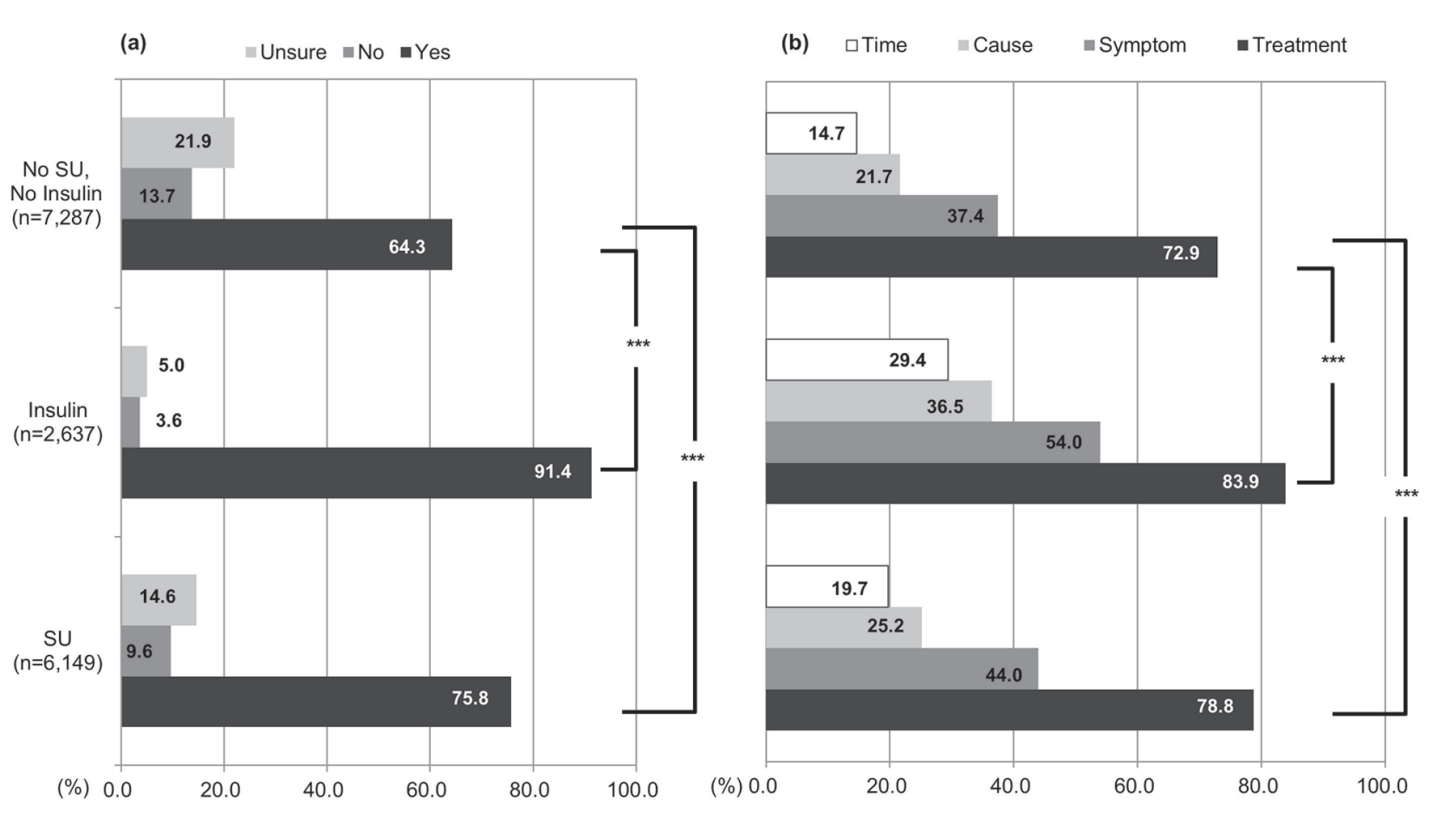
Knowledge of hypoglycemia. (a) “Are you aware of hypoglycemia?” Hypoglycemia awareness in elderly patients who were taking SU, insulin, or neither. (b) “What do you know about hypoglycemia?” Patients who chose “yes” to the first question answered this question. *P < 0.05, **P < 0.01, ***P < 0.001.

Overall, 71.5% patients responded positively to the question, “Do you know what hypoglycemia is?”: 91.4% patients receiving insulin therapy, 75.8% using SU agents, and 64.3% not using insulin or SU agents (Fig. 8). Patients who had used SU agents or insulin had significantly more knowledge of hypoglycemia than those being treated with other drugs (P < 0.001). In the question “knowledge of countermeasures for hypoglycemia”, the majority of patients, with insulin therapy (both monotherapy and combined therapy), with SU agents (both monotherapy and combined therapy), and those not using insulin or SU agents, had high knowledge of countermeasures for hypoglycemia (83.9%, 78.8% and 72.9%, respectively). Although most of patients in the study had some knowledge of countermeasures, more insulin users compared to SU users are aware of the countermeasures. Similarly, 54.0% patients using insulin therapy, 44.0% using SU agents, and 37.4% not using insulin or SU agents had knowledge of symptoms but experienced by hypoglycemia. Unfortunately only 36.5% patients receiving insulin therapy, 25.2% using SU agents, and 21.7% of those not using insulin or SU agents had knowledge of the causes of hypoglycemia. Finally, merely 29.4% patients receiving insulin therapy, 19.7% using SU agents, and 14.7% not using insulin or SU agents had low recognition about times when hypoglycemia was likely to occur.

Regardless of the high level of knowledge regarding hypoglycemia, only 63% patients receiving insulin therapy and 31% using SU agents responded that they always carried glucose or a similar medication with them; this fact confirmed that many patients had knowledge of hypoglycemia but were not executing countermeasures to prevent its onset. Furthermore, we observed that the number of the patients who always carried glucose with them decreased with age; only 49% patients receiving insulin therapy and 21.7% using SU agents carried glucose in the patients aging ≥ 85 years.

## Discussion

In recent years, it has become clear that, when treating diabetes mellitus, hypoglycemia - particularly severe hypoglycemia - causes worsening of the incidence and prognosis of cerebrovascular events [14], and knowing how to prevent hypoglycemia and control diabetes mellitus has gained great attention. In reports dealing with severe hypoglycemia, background factors included advanced age [15], decreased renal function [16], SU agents [17], and insulin use [18]. To prevent such severe hypoglycemia occurring, hypoglycemia should not be overlooked in elderly patients with diabetes during routine treatment, and it is important that proper measures should be taken. The conditions of elderly T2DM patients receiving treatment in Japan, and symptoms and frequency of hypoglycemia remain unclear. Hence, we investigated hypoglycemia based on the results of a survey conducted on the general internists treating patients and the patients commuting to their medical institutions.

### Definition of hypoglycemia in elderly T2DM patients and its relationship with the incidence

In the present study, the incidence of hypoglycemia diagnosed by physicians was 7.8% in the last 1 month and 15.5% in the last 1 year. This frequency is the aggregate of hypoglycemia incidences observed subjectively by primary physicians and that reported by patients; there was no measurement of blood glucose levels, such as the self-monitoring blood glucose levels. There were no strict specifications regarding the rationale for diagnosing the occurrence of hypoglycemia in patients by primary physicians; it was judged based on the subjective opinion of the primary physician. The definition of severity for hypoglycemia is also vague because it was not classified into mild and severe. However, the incidence of hypoglycemia reported in this study was similar to that found in a registry study recently reported in Germany [19]. Targets of the registry studies were patients with T2DM aging ≥ 40 years and using 1 - 2 types of OHAs. The incidence of hypoglycemia during 1 year was 10.5%, as obtained from responses reported by patients [19]. UKPDS73 [18] also showed slightly lower incidence at 11% for 1 year; however, other prospective 1-year registry studies showed an incidence of 14.1% [20]. The authors in the study discussed that it estimated as a lower rate of hypoglycemia in retrospective investigations but the incidence rate was similar to that observed in this study.

Furthermore, the symptoms experienced by most patients during the present study fairly correspond to the symptoms of hypoglycemia in Joslin’s Diabetes Mellitus (14th edition). Therefore, we believe it is sufficiently useful for diagnosing hypoglycemia in elderly T2DM patients.

Notably, irrespective of whether the time period was 1 month or 1 year, there were significant differences in awareness of hypoglycemia between physicians and patients. This suggested the presence of “hidden hypoglycemia that physicians did not detect.” According to Gotoh et al, that is an investigation of patients with diabetes being treated with SU agents or insulin therapy, 49% of patients in whom hypoglycemia occurred did not report the incidence to their primary physician [21]. In Japan, the time to examine a single patient in clinical practice is short and approximately half of these are completed under 10 min [22]. It was also reported that impact of diabetes mellitus on the patients’ lifestyles has not been sufficiently asked [3]; therefore, we believe that it results in differences in the understanding of hypoglycemia between physicians and patients. We suggest that it is important to confirm the presence of hypoglycemia by focusing on asking regarding definite symptoms when examining patients who are susceptible to hypoglycemia during each examination.

In addition, it must be noted that elderly patients with diabetes may describe a sign or symptom of hypoglycemia as general tiredness during physical examination. Even during the present study, in addition to symptoms such as “back pain” and “frequent urination,” which were believed to be due to geriatric syndrome, symptoms of general malaise such as “cramps”, “numbness in the legs”, and “swelling” and “easy to get foot cramps” appeared frequently. A relationship between diabetes mellitus/hypoglycemia and symptoms such as “cramps” has been reported [23, 24]; therefore when asking about hypoglycemia during routine examination, physicians should inquire patients regarding not only the known conventional symptoms of hypoglycemia but also the various symptoms of general malaise shown that many patients have in this study.

Normally, the most frequent symptoms of hypoglycemia are symptoms caused by autonomic nervous system, such as “sweating” and “palpitations,” and the most common central nervous system symptoms, such as “confusion,” “lightheadedness,” and “malaise” [25]. However, in patients aging ≥ 70 years, central nervous system symptoms of mild headache and unsteadiness are reported to be common [26]; the present study also showed that the OR of the symptoms originating from the sympathetic nervous system was low, and the parasympathetic symptoms were ranked higher in the autonomic symptoms.

In addition, the multivariate analysis for symptoms experienced by patients in 1 month and symptoms of hypoglycemia, OR of central nervous system symptoms of hypoglycemia listed in Joslin’s Diabetes Mellitus (14th edition), i.e., “forgetfulness” and “headache,” were low. We consider that this is because the patients had poor knowledge that the symptoms such as “forgetfulness” and “headache” are hypoglycemic symptoms. Therefore these can be “hidden hypoglycemia because of the patients’ lack of knowledge.” It is important that the physicians instruct their patients to take glucose immediately when an atypical hypoglycemic symptom appears and confirm whether a symptom is improved.

The incidence of hypoglycemia does not increase when HbA1c decreases, rather hypoglycemic risks increase when HbA1c values rise. The incidence of hypoglycemia was investigated in 7,855 patients using SU agents or insulin; the incidence of hypoglycemia increased in both the groups with low and high HbA1c. Other studies have also reported that the risk of hypoglycemia increases in patients with poor glycemic control [27, 28], and notably, hypoglycemia needs to be taken into account in elderly Japanese T2DM patients who have high HbA1c values. In addition, it is known that the risk of hypoglycemia is typically higher in patients with a long disease duration or those with diabetic complications, including young patients [29]; similar results are also found in the present study. We believe closer attention needs to be paid to hypoglycemia in elderly patients with lower BMI and poor glycemic control.

### Relationship between hypoglycemia and antidiabetic drugs

Similar to previous studies [30, 31], insulin was associated with an exceptionally high incidence of hypoglycemia, and the incidence was high in patients using SU agents or glinides as OHA monotherapy. The risk of hypoglycemia for patients using DPP-4 inhibitors was the smallest. Similar trends were found in analysis with combined therapies, a strong association with not only insulin but also SU agents and glinides, a low risk of hypoglycemia when using DPP-4 inhibitors. This shows that DPP-4 inhibitors are effective at preventing hypoglycemia and have a lower incidence of adverse events than other OHAs [32]. In addition to these safety aspects, there is abundant useful evidence for their use in elderly T2DM patients as they improve symptoms of dementia [33] and bone metabolism [34]. It is why DPP-4 inhibitors are commonly used as first-line treatment.

Although SU agents have a high risk of hypoglycemia, 34% of patients even with HbA1c ≤ 7% used SU agents in the present study, indicating that these patients are with a latent risk of hypoglycemia. The average dose of SU agents is comparatively low in the present study; however, a report has suggested that even a small amount of SU agents could put elderly patients at risk of experiencing hypoglycemia [35]. For this reason, great attention is required when managing hypoglycemia, even at low doses. Furthermore, even DPP-4 inhibitors, its use combined with SU agents could cause severe hypoglycemia, and also reported that the incidence frequency of severe hypoglycemia changes based on the type of SU used in the combination [36]. Among the SU agents, compared with glibenclamide or glimepiride which have a high affinity for SU receptor on the pancreatic B cells, gliclazide which has a long half-life and a weak affinity for the receptors has been reported to show low frequencies of mild and severe cases of hypoglycemia; however, this tendency was not observed in the present study. This suggested the requirement for caution when using any kinds of SU agents in the elderly. In practice, as shown in other epidemiological studies, the frequency of SU agents use decreases with time [37]; however, the use of SU agents is frequently used concomitantly in Japan, and attention is thus required on the effects of any combination use.

### Knowledge of hypoglycemia

We found that patients using insulin and SU agents have a higher level of knowledge of hypoglycemia than patients using other drugs. This is considered to be because many patients have experienced hypoglycemia due to their trends that these drugs readily cause hypoglycemia, and have been frightened by the experience [38]. However, there is an investigative report stating that patients do not proactively notify their physicians when hypoglycemia occurs [21]; another report has stated that primary physicians have insufficient knowledge regarding patients’ hypoglycemia [39]. Particularly among routine medical treatments, it is important to ask them regarding hypoglycemia during each visit, when patients are using insulin and SU agents.

Even among the patients who answered that they “had not experience hypoglycemia,” many patients presented with hypoglycemic-like symptoms, and it is likely that the number of patients who have actually experienced hypoglycemia is higher than the number. This also suggested the presence of “hidden hypoglycemia that patients were not aware.” Normally, glucagon and adrenaline, the so-called anti-insulin hormones, are secreted when the blood glucose levels decrease and the autonomic symptoms occurring due to secretion of adrenaline may serve as a warning sign for hypoglycemia. At this point, prevention of severe hypoglycemia is possible if it is noticed and glucose is ingested. If it is not done promptly, the blood glucose levels will decrease further until central nervous system symptoms, such as decreased cognition, appear and it becomes difficult to treat the hypoglycemia, and the possibility of becoming severe hypoglycemia is high. In elderly patients, in particular, central nervous system symptoms such as slurred speech and incoordination appear more readily than autonomic symptoms [26], and the blood glucose threshold for appearance of autonomic symptoms is lower compared with younger patients. On the other hand, the threshold for blood glucose level for the appearance of central nervous system symptoms is higher in elderly patients [40]; therefore, this is one factor that delays the appropriate management of hypoglycemia. With advanced age, the ability to recognize hypoglycemia weakens [4], and there are some cases where the patients themselves are unaware of hypoglycemia. Acquiring knowledge regarding hypoglycemia reinforces subjective perception of hypoglycemia [41], and it is important to raise awareness of hypoglycemia to prevent serious situations.

In the present study, the degree of knowledge regarding hypoglycemia was comparatively good overall, but when we examined the details, frequency of patients who knew “what time periods were associated with the appearance of hypoglycemia” was low. The response to questions indicating preparedness for hypoglycemia, such as “Do you have glucose on you?” was lower than expected in patients using SU agents, and the tendency was more common as the patients aged. Thus, during routine examination, the physicians should regularly provide guidance regarding how to manage hypoglycemia, and it is also important to actually hand glucose to patients. It may be possible that elderly patients cannot understand the types of diabetic medication that was prescribed to them, although a survey item for it was not included in the present study. In any case, the treatment should be explained to patient themselves and a key member of the family, and basic guidance should be provided regarding the methods of managing hypoglycemia; this guidance should be continuously repeated in the future.

### Conclusion

The results of the present study suggested the presence of two types of “hidden hypoglycemia”, namely: “hidden hypoglycemia that physicians did not detect” and “hidden hypoglycemia that patients were not aware.” Compared with younger patients, elderly hypoglycemic patients more commonly have atypical symptoms of hypoglycemia, and the hypoglycemia is not noticed in the first place. These conditions also promote the development of severe hypoglycemia [4]. In addition, consistent hypoglycemic symptoms do not necessarily appear in the same patient [42]. Further investigation of the relationship between hypoglycemia and various symptoms in elderly T2DM patients, including unidentified complaints, should be encouraged. In addition to establishing a highly precise tool enabling easy diagnosis of the appearance of hypoglycemia in patients by the family physician, the patient should also be proactively inquired regarding whether they have such symptoms. It is vital that physicians strive to prevent overlooking hypoglycemic symptoms. Furthermore, instructions should be provided to the patients regarding glucose intake when these symptoms appear. The patients’ own degree of understanding and readiness for hypoglycemia must be important for its prevention.
